# Editorial: Women in neurology: sleep disorders

**DOI:** 10.3389/fneur.2026.1901855

**Published:** 2026-07-29

**Authors:** Athanasia Pataka

**Affiliations:** Respiratory Failure Unit, Medical School Aristotle, University Thessaloniki Greece, G Papanikolaou Hospital, Thessaloniki, Greece

**Keywords:** gender differences, health equality, neurology, personalized medicine, sex differences, sleep disorders, sleep medicine, women's health

Sleep disorders affect millions of individuals globally and impair neurological health, cognition, and mood regulation, cardiovascular and metabolic function. Despite the fundamental role of sleep and its effects in several aspects of health, women have been under diagnosed or mischaracterized in several sleep disorders and additionally have been underrepresented in scientific leadership. The collection of Frontiers “*Women in neurology: sleep disorders*” was designed to highlight research led by women scientists in sleep medicine and neurology. The articles included in this Research Topic collectively highlight the multidimensional nature of sleep medicine, incorporating epidemiological observations, clinical implications, and translational perspectives. This Research Topic, serves as a scholarly contribution to sleep medicine, but also as a statement on the importance of female's visibility, leadership, and equity in neurological research for sleep medicine.

Recent research increasingly demonstrates that women frequently exhibit distinct symptom profiles, hormonal influences, and clinical trajectories in sleep disorders, including insomnia, obstructive sleep apnea (OSA), restless legs syndrome, and circadian rhythm disorders. Therefore, this Research Topic is particularly timely. The paper of Perger et al. provides a comprehensive overview of sex-related differences across major sleep disorders. Women frequently exhibit atypical symptoms of OSA, including fatigue, depression, insomnia, or mood disturbances, rather than the classic symptoms more commonly reported in men. This distinction carries significant clinical implications, as insufficient recognition of female-specific phenotypes may result in delayed diagnosis and inadequate treatment. Experimental animal models have elucidated hormonal and neurobiological mechanisms underlying sex-specific sleep regulation. These findings are subsequently linked to clinical observations concerning symptom expression, disease burden, and therapeutic adherence (Perger et al.). Through the integration of basic science and patient-centered clinical research, an increasing sophistication of gender medicine within the field of sleep neurology is demonstrated recognizing sex- and gender-related biological differences as key factors in disease expression, diagnosis, and treatment.

Another important contribution of this Research Topic is its emphasis on interdisciplinary collaboration. Sleep disorders do not exist in isolation; they interconnect with pulmonology, neuroscience, psychiatry, endocrinology, cardiology and dentistry. The featured studies and reviews demonstrate the need for integrated models of research and care in different fields and periods of women's life. This interdisciplinarity is particularly relevant in women's neurological health, where symptoms are often multifactorial and influenced by hormonal factors, psychosocial stressors, caregiving burdens, and disparities in healthcare access. The review of Vaienti et al. emphasizes that the diagnosis of sleep disorders should not be delayed, as early recognition and treatment are essential for preventing adverse health outcomes even from childhood. It is therefore important that healthcare professionals across multiple specialties, including dentists, pediatricians, and primary care physicians, are familiar with the clinical presentation, risk factors, and potential consequences of sleep disorders to facilitate timely identification and appropriate referral (Vaienti et al.). Equally, the development of educational initiatives to ensure clinicians recognize gender-specific manifestations of sleep disorders in routine practice is important. The study of Arcidiacono et al. demonstrated that there is a lack of knowledge about pediatric OSA and its management among Italian dentists, revealing the need to update the dentistry curriculum and organize educational interventions.

Sleep quality is essential for wellbeing and is affected by socio-economic and cultural factors. In the review of Olana et al., nearly half of Ethiopian women of reproductive age were found to experience poor sleep quality. Key contributing factors for poor sleep quality included stress, unplanned pregnancy, substance use history, intimate partner violence, depression, and co-morbid conditions. This highlights the importance of timely identification of at-risk individuals through targeted screening, preventive strategies and implementation of effective interventions to improve sleep quality especially in venerable populations (Olana et al.). Self-management interventions may offer significant benefits for women experiencing sleep disturbances by providing practical strategies to reduce stress and enhance sleep quality, both of which are critical for overall health and daily functioning. Among non-pharmacological strategies, Global Postural Re-education (GPR) has emerged as a promising intervention. GPR utilizes active and progressive postures to stretch muscle chains, improve muscle coordination, optimize breathing patterns, and enhance proprioceptive awareness. In the study of Rodríguez-Aragón et al., GRP facilitated stress reduction, promoted physical wellbeing, and improved sleep quality, here in female health science students.

Different periods of women's life, as puberty, pregnancy, perimenopause, and menopause, significantly affect sleep architecture and susceptibility to sleep disorders. The review of Zhang et al. demonstrated that gynecological conditions, as endometriosis, are related to sleep disorders. Sleep disorders in patients with endometriosis are multifactorial and result from the interplay of physical, psychological, and biological factors. Chronic pelvic pain, dysmenorrhea, and dyspareunia can disrupt sleep by causing discomfort and frequent awakenings, while infertility, miscarriage risk, and concerns about associated chronic diseases contribute to emotional distress, anxiety, and depression. Additionally, hormonal and inflammatory dysregulation, chronic fatigue, reduced physical activity due to pain, and the potential effects of medications used to treat endometriosis may further impair sleep quality. These factors place women with endometriosis at a substantially increased risk of developing sleep disturbances and poor sleep quality (Zhang et al.).

In addition, this Research Topic of articles reflects broader methodological evolution within sleep research. Advances in neurophysiology, polysomnography and biomarker discovery are reshaping how clinicians and scientists conceptualize sleep pathology. For example, in the study of Lin et al. patients with insomnia disorder were found to have significantly higher serum Glu levels and increased NMDAR NR1 subunit mRNA expression, suggesting dysregulation of glutamatergic neurotransmission and supporting further investigation into glutamatergic mechanisms and their potential as diagnostic or therapeutic targets in insomnia disorder. Furthermore, the study of Cai et al. demonstrated that reduced serum levels of DAO, D-lactic acid (D-LA), intestinal fatty acid-binding protein (I-FABP), and endotoxin (ET) were observed in insomnia patients reflecting possible long-term alterations in intestinal barrier function.

In conclusion, this Research Topic highlights scientific advances and the importance of promoting women's leadership in sleep medicine. These investigations are critical, as conventional diagnostic paradigms for sleep disorders have primarily been established using male cohorts, which has led to delayed recognition and under-treatment of women. By focusing on sex-specific perspectives, this Research Topic emphasizes the need for individualized care, increased diversity in research, and recognition of biological and gender-related differences. These efforts support more equitable and comprehensive healthcare.

